# Randomised trial reveals a mismatch between preferences for and hormonal responses to anthropogenic light colour temperatures

**DOI:** 10.1371/journal.pone.0327843

**Published:** 2025-08-11

**Authors:** Solène Guenat, Jörg Haller, Nicole Bauer

**Affiliations:** 1 Social Sciences in Landscape Research, Economics and Social Sciences, Swiss Federal Institute for Forest, Snow and Landscape Research, Birmensdorf, Switzerland; 2 Elektrizitätswerk des Kantons Zürich, Zürich, Switzerland; Federal University of Paraiba, BRAZIL

## Abstract

Public streetlights are universally used to improve visibility after dark and improve residents’ safety. However, anthropogenic light negatively impacts human health and well-being, biodiversity and energy consumption. Anthropogenic light impacts could be mitigated by technological changes optimising light characteristics, yet we know little of light colour temperature’s influence on well-being. Here, we aim to examine the impact of exposure to LED streetlights of 2700K, 4000K and 6500K on the impression of light, the feeling of safety, and the well-being (affect, self-reported stress and physiological stress). We used a parallel group field experiment with 77 participants, over 18 years old, in a small Swiss town with controlled light settings. Participants were randomly allocated to a light treatment through computer-generated randomisation. With 25–26 participants per treatment, we showed that participants had better impressions of warmer temperatures than of cold ones. Light temperatures did not influence affect, the feeling of safety or self-reported stress, yet the decrease in cortisol was stronger under 6500K than under 2700K. The observed lower hormonal stress levels in 6500K lights can be attributed to their resemblance to daytime light temperatures, while preferences for warmer lights reflect the expectations for night-time situations.

## 1. Introduction

### 1.1. Anthropogenic public lighting within the urban sustainability debate

Urban sustainability requires a fine balance between, among others, having low energy consumption, decreasing the negative impacts of urban areas on biodiversity and promoting health and well-being for the population [1]. No issue of urban planning exemplifies this conundrum better than anthropogenic light at night (ALAN). Public lighting is considered positive for sustainability due to the perception that an illuminated city is safer, more attractive and providing more opportunities for economic activities [2]. However, public lighting also have negative impacts of urban sustainability: it is responsible for up to 50% of municipalities’ energy consumption [3] and negatively influences biodiversity as well as human health and well-being by, e.g., impairing reproduction and development, perturbing the sleep and activity rhythms, disturbing species movement and trophic interactions, and elevating risks of cancer [4–6]. Consequently, there is an urgent need to find mitigation measures allowing for sufficient public lighting for a high feeling of safety while decreasing its negative impacts, for instance by adjusting light characteristics such as energy-consumption, intensity or correlated colour temperature (CCT).

Impacts of ALAN characteristics on biodiversity and energy consumption are relatively well documented. For instance, energy consumption increases with intensity and white light-emitting diodes (LED) lamps are up to 15 times more energy-efficient than orange high-pressure sodium (HPS) lamps to reach the same illumination [7]. A transition to LED could thus decrease energy consumption and related economic costs while maintaining the same light intensity, yet at the expense of colours becoming colder and with the risk of a rebound effect in which public lighting use would increase [8]. Changes in light CCT also affects health through the melatonin cycle [9]. As for biodiversity, warmer CCT are less detrimental to night-flying insects than whiter ones [10–12], though there seem to be no impact on either bats or birds [10,13].

### 1.2. Considering well-being in urban (light) infrastructure planning

Well-being of the population is one of the main aims for urban sustainable development, and can be reached “through environmentally sound urban and territorial planning, infrastructure, and basic services” [1]. Well-being is a multidimensional construct defined as “a state of happiness and contentment, with low levels of distress, overall good physical and mental health and outlook, or good quality of life” [14], though other definitions also focus on social connections, spiritual meanings, physical health or activities and functioning [15]. There is growing evidence that, though mental well-being is in part determined by individual, biological and socio-economic factors [16], it is also strongly influenced by environmental conditions. For instance, well-being is positively impacted by urban greenspaces [17] and biodiversity [18,19], or negatively impacted by noise [20] or general anthropogenic light levels [21].

Impacts of anthropogenic light on human well-being are increasingly documented on a large-scale level, with overall levels of ALAN known to harm human health and well-being [22,23]. Yet evidence on the impact of different outdoor anthropogenic light’s characteristics at the local level focus mostly on intensity, with stronger levels of intensity associated with increased feeling of safety [24], hypothesised to lead to a better well-being. Evidence on the impact of different CCT on well-being however remains scarce and patchy.

### 1.3. Direct impacts of CCT on well-being

In studies on outdoor anthropogenic light, well-being is mostly measured through the affect, namely “any experience of feeling or emotion” [14], or through self-reported stress levels. In a virtual environment, a CCT of 4500K triggered less tiredness, sadness and nervousness than either 2500K or 6500K [25]. However, among the negative emotions triggered by 2500K lights, tiredness was more important than sadness [25]. Studies in indoor settings showed that blue-enriched white light of 17’000K led to higher ability to concentrate and more positive affect as compared to exposition to both 4000K [26] and 2900K [27], in particular by improving alertness. Similar higher levels of alertness and positive affect in whiter lights were found with smaller differences in CCT, comparing 6500K with 3500K [28].

Preferences for specific environmental characteristics have long been described as expressions of our underlying unconscious need for well-being [29]. This has been substantiated by studies showing either a linear relationship between perceived restorativeness of a greenspace and preferences for such greenspace [30] or that landscape preferences align when expressed in general terms or with the specific aim of stress relief [31]. In term of outdoor light CCT, there is no consensus as to the preferences of the population. Several studies evaluating the impression of street-scale illumination found that people had a better impression of warmer CCT, irrespective of whether they were emitted by HPS or by LEDs [32,33]. These preferences were also valid for people with visual impairment [34]. Yet others highlighted that newly installed white MH lights were found more attractive and having a more pleasant glow than formerly used warmer HPS [35]. Large-scale surveys also highlighted that yellow light were more likely to be perceived as unpleasant, in particular when colouring snow [36], and that < 3000K lights were perceived as unsatisfactory as opposed to >3000K lights [37]. Several studies both outdoor and in lab experiments also showed equal appreciation of light across CCT [38,39]. Some studies also highlight a mismatch between how CCT influences the perceived light quality, including its influence on the ability to orientate oneself, and the perceived comfort. While the light quality is rated higher in white CCT, there is a higher perceived comfort in warm CCT [34,40]. Understanding, in a standardised environment, the preferences of residents of a small European town for different light CCT would thus greatly help install light CCT that align with the wishes of the population, while respecting their well-being.

### 1.4. Indirect impacts of CCT on well-being

ALAN CCT can also influence well-being through indirect pathways. Firstly, one of the initial reasons for widespread anthropogenic light implementation is the belief that ALAN improves the safety of urban residents [3]. Although the relationship between crime and artificial lighting is context-dependant [41,42], increasing ALAN levels consistently decreases the number of traffic accidents with injuries [43]. Additionally, the perception that one environment is safer with light might in itself be sufficient for people to be influenced by the light conditions in selecting the use of different environments [44]. In the built environment, the feeling of safety is theorised to be influenced by the point to which one can (1) have a good overview of the environment (prospect), (2) perceive the environment to provide hiding places for potential criminals (refuge), and (3) escape from potential threats (escape) [45]. Artificial light can influence these relationships by altering the overview or the perceived hiding places through the spatial distribution of the light [46] or through its uniformity [44]. We found no studies investigating how CCT influences the overview or the perceived hiding places. Irrespective of the prospect-refuge-escape theory, the relationship between the feeling of safety and CCT is unclear. Within the same study, evidence points towards Spanish urban residents showing no differences in how they feel towards the risk of suffering from criminal activities based on CCT, while they perceive lower risks of accidents in white (MH or LEDs) than in yellow-sodium lights [32]. This lack of differences based on CCT is consistent how white MH and yellow HPS influence the feeling of safety in parking lots [38]. Differences are identified by other studies, yet not always in the same directions: whereas whiter CCT are associated to higher feeling of safety in US students walking in forested peri-urban areas [47], Chinese residents of urban areas feel safer in warm and uniform lights [37]. CCT can also influence how bright the light is perceived [32,38,48], which in turn influences the feeling safety [24].

Another indirect pathway through which light characteristics could influence well-being is through stress reduction. Stress is “a state of worry or mental tension caused by a difficult situation” [49] and a natural response to challenges, which might be coming from the characteristics of the environment. Although a short-term measure of worry, stress is highly related to well-being [50]. High positive affect can also help mitigate stress by increasing resilience and buffering negative impacts [51]. The attention restoration theory states that people can concentrate better and reduce their stress after a time in natural environments as these environments contain characteristics which help restore the capacity for directed attention [29]. Even though artificial light is not part of the natural environment, it can be tuned to be reminiscent of natural light conditions or highlight natural features, thus triggering stress-alleviating restorative experiences and leading to more positive affect. For instance, in a computer simulated illumination with white MH halide lamps, Nikunen and Korpela [52] found that a focus of the light on greenery lead to a more stress-alleviating, restorative experience. In addition to focus, light characteristics could be modified for intensity or CCT to mirror daytime sunlight conditions, e.g., a CCT mostly between 5400K and 6250K [53]. Any CCT outside this range could be perceived as “unnatural”. In the only identified outdoor cross-sectional study on CCT, no difference in self-reported stress levels was found between yellow HPS lamps and white metal-halide (MH) or LED lamps [32]. Although exposure to indoor anthropogenic light of different characteristics such as intensity or CCT is known to impact hormonal regulation, including the stress hormone cortisol [28,54], there is a lack of knowledge on the influence of outdoor CCT. In indoor environments, the evidence is mixed. Lower cortisol levels were induced by 9000K blue-enriched lights as opposed to 2800K warm light, in particular for over-55 years old [55]. Conversely, Choi et al. [28] found no significant differences in salivary cortisol concentrations after exposure to 6500K or 3500K lights, and that despite an impact on melatonin [28].

### 1.5. Current study

Consequently, there are suggestions that CCT can be key to well-being of urban residents and that indiscriminately installing white LEDs for their energy efficiency might not provide the best output in term of sustainability. However, we still lack a clear picture of which CCT provide the most well-being benefits, be it as a direct impact on affect or impression, or an indirect one through improving the feeling of safety or reducing stress levels.

Against this background the present paper aims to answer the following research questions through a field experiment:

- What is the impact of exposure to streetlamps of different CCT on affect, self-reported stress and cortisol levels?
- Does the impression of artificial light and of the feeling of safety provided by streetlamps differ based on its CCT, and if so, how?

In a small-town centre in Switzerland, we used a randomised trial in field experiment with controlled light settings to assess the differences in impressions, affect, stress and the feeling of safety, based on self-reports and hormonal measurements, according to light correlated colour temperature from warm to cool white. We hypothesis that there will be differences in impression, affect, stress level and the feeling of safety based on CCT, with the warmer colour temperature providing a preferred and more restorative environment, while the cooler colour temperature provides a higher feeling of safety.

## 2. Methods

### 2.1. Study site

Switzerland is localised in central Europe and is remote from any zone totally free of anthropogenic light [56]. Its levels of light emissions are relatively low compared to other countries in the Global North [56] though the country experiences an important geographical variability in light emissions as the alpine regions, which cover about 60% of the territory, emit less than a third of the country’s anthropogenic light [57]. In a regulatory point of view, the Swiss Federal Office for the Environment recognises light pollution as problematic and issued non-binding recommendations to the municipalities for preventing light emissions [58]. Within Switzerland, we used the local case study of Richterswil (ZH) to evaluate the impact of CCT through a field experiment. Richterswil was selected as a case study for two reasons. Firstly, it a typical small town in Switzerland, as it aligns with the Swiss population in terms of age and gender structure, migration background of the population, working population, and architecture [59–61]. In 2022, it had a population of 13’966 inhabitants, 51.7% of whom were women [60], compared to the 50.4% throughout the Swiss population [59]. The average age was 43.5 years old in Richterswil [60], and 42.6 years old in Switzerland [59]. The working population mostly commutes to the neighbouring municipalities and to Zürich and the unemployment rate of 2.1% aligns exactly with that of Switzerland [60]. The town centre has a population density of 80–160 inhabitants per square meters and most of the buildings date from the 1600-1800s, with a few additions in the 1990s [61]. Secondly, the town inaugurated in 2022 the revitalisation of its centre, including its transformation into a 20 km/h pedestrian zone in which benches with vegetation and new luminaires were installed [62]. These luminaires offer the possibility to change CCT, and thus provided the rare technical opportunity to use different light colours in the same public space.

### 2.2. Study design

**Fig 1:**
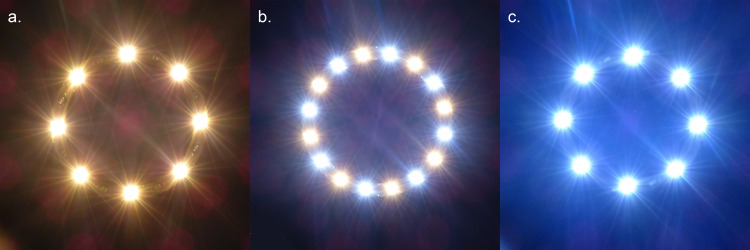
Luminaires seen from below. The different light colours were set up by turning on (a) only the 2700K LED; (b) both the 2700K and 6500K LED to achieve a 4000K colour; and (c) only the 6500K LED. Luminaires were not looked at directly by the participants.

**Fig 2:**
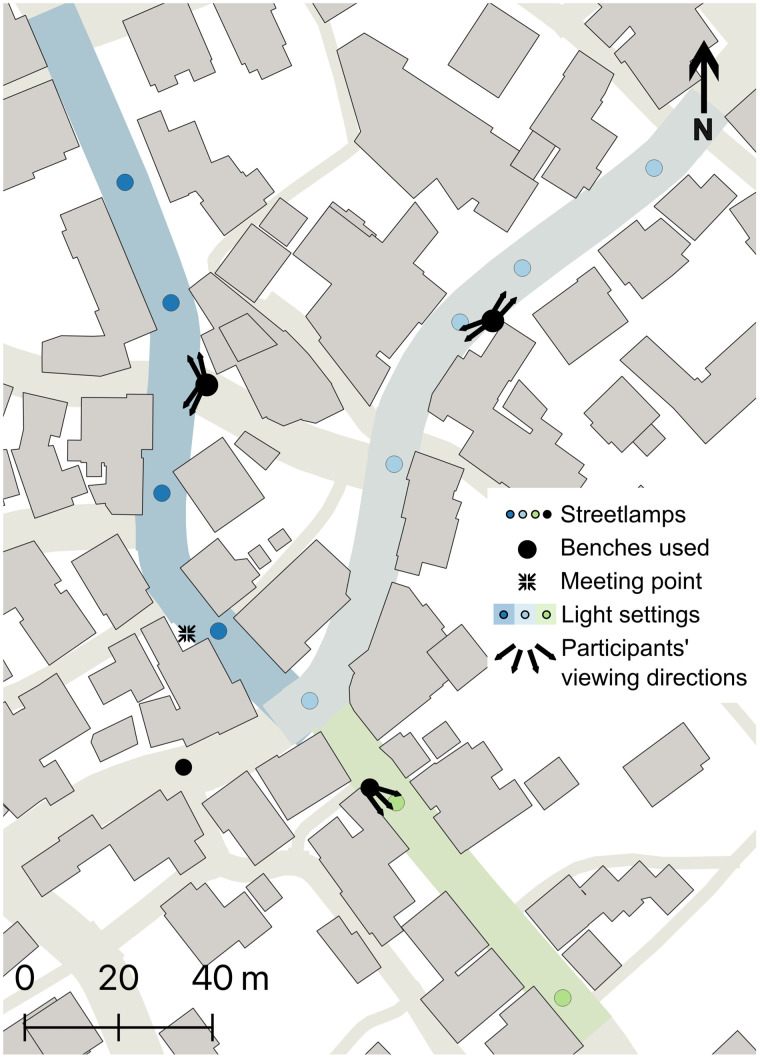
Map of the study site in Richterswil centre. All coloured roads are fitted with streetlamps with variable colour and intensity settings. Thanks to the buffer area and corner, none of the sectors are visible from another. Basemap: Swiss Federal Office of Topography swisstopo.

**Fig 3:**
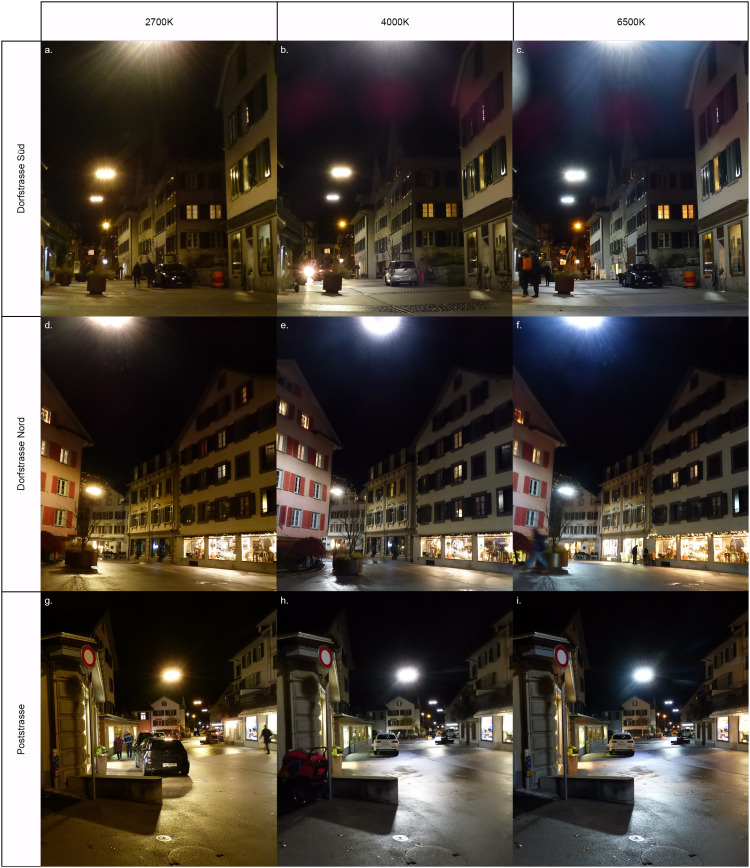
The three street segments illuminated with the three different CCT. **(a-c)** Dorfstrasse Süd, green on Fig 2; **(d-f)** Dorfstrasse Nord, dark blue on Fig 2; **(g-i)** Poststrasse, light blue on Fig 2. **(a, d, g)** 2700K, **(b, e, h)** 4000K, **(c, f, i)** 6500K.

We used a randomised intervention study and a longitudinal, between-subjects and parallel group design. We used three light treatments differing in terms of CCT. Light colours included were: 2700K (warm), 4000K (white) and 6500K (cool white). This roughly aligns to CCT gradients used in previous studies on the perception of anthropogenic light colour [32,25,63]. The public was not included in the trial design. The lights used in the experiment were specially developed and built for this situation and consisted in a round structure with 16 different LED-arrays, equally split between either 2700K or 6500K. The lighting system was also equipped with a special control system that enables all light colours to be controlled continuously. It is the first known system of its kind in the Swiss public space. The luminaire was developed by and in co-operation with company Bergmeister from Germany. Commercially available LEDs were used. The use of a combination of either lightbulb allows for different light colours (Fig 1). The light treatments had different energy consumptions, with the 6500K having a 30% lower energy consumption than the 2700K, and the 4000K a 15% lower energy consumption than the 2700K. The luminaires were hanging in the middle of the street, at a height of about 14m. This differed from the light colours used outside of the experimental period, which consisted of a gradual transition from colder to warmer colours on the course of the evening. By dividing the study site in three street segments not visible from each other (Fig 2), we could decrease the influence of day and time by presenting the three different treatments, to different participants, simultaneously in different streets. Similarly, as each treatment was allocated to the same number of participants in each street segment, the impact of other light sources or other disturbances (e.g., noise, presence of shops) specific to each street was mitigated (Fig 3). Light intensity was consistent across the three CCT, with an average luminous flux (ground illumination) of 7.5 lx + /- 10%.

We used a computerised random number generator to randomly assign each participant to one of the three treatments and street segments. Randomisation was simple, given the low sample size, with the only restrictions being that participants registering in groups (e.g., couples) were not assigned to the same session (treatment or timeslot). Participants were not made aware of which treatment they were allocated to, although the visual differences between the treatments could reveal it. However, contrarily to a typical randomised control trial, all treatments differed in some way to typical street illumination, reducing the risk of bias. Participants were exposed for 20 min to their specific treatment, during which they stayed seated on public benches, without looking directly at the streetlamps. Twenty minutes was selected as an appropriate time as people stay on average 15 min in the street environment [64] and the peak of cortisol takes place about 20 min after exposure [65]. Before and after exposure, participants filled a questionnaire (S1 File) and gave salivary samples. There was no further follow-up after the post-exposure questionnaire and salivary sampling. Researchers were present at the meeting point at all times to supervise the experiment and answer any question by the participants.

The study took place from Nov 1 to Nov 20, 2023, while the night was falling relatively early and there were no Christmas lights installed. Two sessions took place per evening, either from 18:30 or from 20:30. Those times were selected because that is when people are most likely to be outdoor at night-time [66]. Averaged air temperatures during the one-hour of the experiment varied between 2.99°C and 13.77°C (mean = 7.16; SE = 4.27; Table A in S1 File) [67]. Though most sessions did not experience any precipitations, there were an average of 0.17 mm (SE = 0.25) of rain [67]. Wind speeds measured at the nearby weather station were relatively low, with an average (+/-SE) of 8.44 (+/-2.92) m/s, and the sheltered city environment means that the wind speed perceived during the experiment was likely lower [67]. Relative air humidity varied between 95.03% and 54.10% (mean = 82.77; SE = 27.31) [67].

This study was approved by the ETH Zürich Ethics Commission on September 04, 2023, a process including a full review of the study protocol, and the statistical analysis plan has been discussed with the ETH statistical consulting service. Written informed consent to use salivary samples and questionnaire data for research purposes was obtained from all participants prior to the experiment.

### 2.3. Participant selection

Participants were invited by sending information flyers to 5000 out of the 5333 households of Richterswil, advertising the study through the UZH Marktplatz, through the WSL mailing list and through friends and family. Recruitment started on Oct 6 and ended on Nov 12, 2023. Participants were eligible if they were above 18 years old. Criteria for ineligibility were total blindness, pregnancy, Cushing’s syndrome, BMI over 35 or intake of cortisone. Participants were free to select the day and time-slot at which they would take part in the study, leading to different number of participants per session. Consequently, participation took place in groups of one to three person per treatment, with a maximum of nine persons taking part simultaneously. Final sample size was not pre-calculated but determined by the number of volunteers after having maximised the sampling effort.

### 2.4. Outcomes

#### 2.4.1. Impression of the light.

We evaluated the general impression of the streetlamps CCT and intensity in the street through 7-points rating-scale questions (Section 2 in S1 File). We also used questions about the general impression of the light and whether the light was disturbing [32]. Additionally, we asked an open question about whether the participants had comments about the lighting of the street. The general impression was only evaluated post-exposure.

#### 2.4.2. Feeling of safety.

We measured the general feeling of safety through an adapted version of the perceived personal danger scale [68] already used in the context of anthropogenic light [69], and through two questions on the perceived impact of lights on accidents [32]. As there is to our knowledge no validated scale to assess the prospect-refuge-escape dimensions of the feeling of safety, we measured them through two questions previously used with this aim [68]. The feeling of safety was only assessed post-exposure.

#### 2.4.3. Well-being.

We assessed the well-being through the Positive and Negative Affect Schedule (PANAS) scale and stress levels. The PANAS consists of 20 adjectives to be rated according to how participants feel [70]. Adjectives for the positive affect include, e.g., active, excited, inspired or proud, whereas those for the negative affect include, e.g., distressed, irritable, jittery or hostile. This scale has been validated while covering different time-periods, and its applicability to assessing the present mood has been assessed and considered responsive to external factors [70]. The German translation of the PANAS scale that we used has been validated [71]. We used this scale with a repeated assessment pre- and post-exposure.

We measured stress using two different methods. We used salivary cortisol, a common biomarker for stress reactivity [72]. The salivary cortisol was collected with cotton swaps kept in the mouth for 2 min. To avoid contamination, participants were instructed not to drink alcohol for the 12h preceding the experiment, and not to eat, drink or take medication in the hour before. For their analysis, we requested a duplicate Cortisol immuno-assay to Dresden LABservice GmbH (Germany). We also used a self-report of stress level through a single question, namely “please select how stressed you feel right now”, with a 7-points rating answer options, labelled at the extremes from “not stressed at all” to “extremely stressed”. Both measures took place pre- and post-exposure.

### 2.5. Explanatory variables

In addition to light colour, we included gender and age class (in 10-year groups, as a discrete variable) as socio-demographic confounding variables. We also explored, as confounding variables, the role of the street the participants sat in, the weather they experienced during the experiment (as a categorical variable being either rainy or not) and the vision of the participants (whether or not they wore glasses or contact lenses during the experiment). It is indeed known that people without glasses tend to find artificial streetlamps darker and less comfortable than those wearing glasses [38].

### 2.6. Data preparation

**Fig 4:**
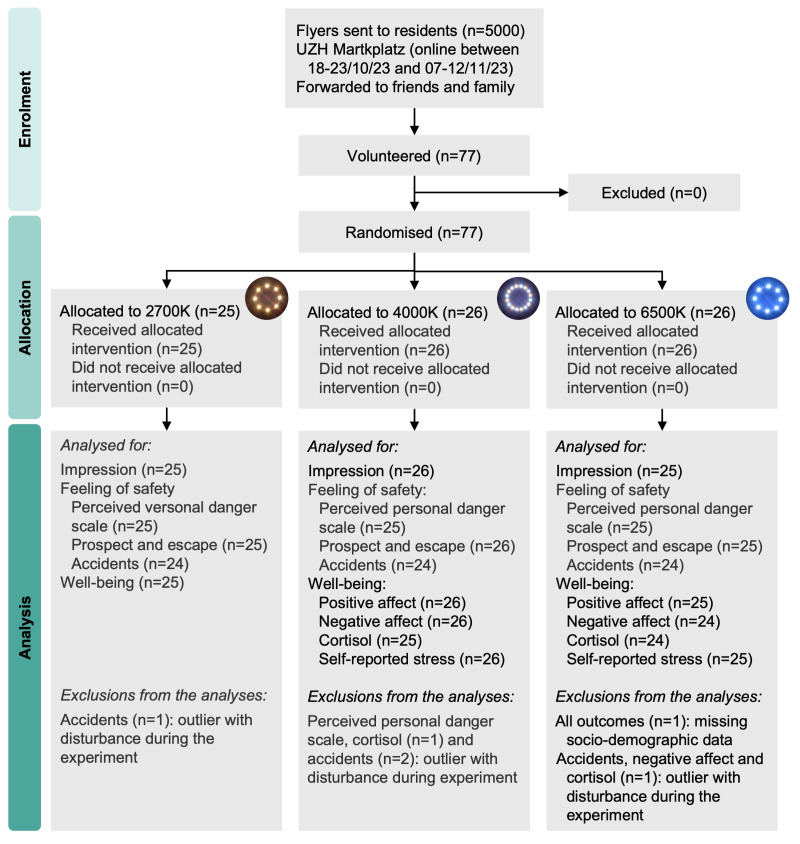
Participant flow diagram with number of included and excluded participants.

Following guidance on PANAS analysis [71], we averaged all values from the positive affect and all values for the negative affect. We aggregated through additions the items of the perceived personal danger scale and the perceived impact of light on accidents. All other values from the questionnaires were kept as ratings. We excluded one respondent allocated to 6500K due to missing socio-demographic data (Fig 4). Data was double-checked for outliers for each outcome, and participants were excluded from the analysis in question if they were below the first or above the third quartile and had experienced disturbances. Disturbances included having been verbally accosted in a threatening manner during the experiment (n = 1), having had acquaintances passing during the experiment (n = 1) or having experienced a technical issue leading to a shorter segment of the street being of the correct colour (n = 2).

### 2.7. Statistical analysis

Depending on the structure of the data, the outcomes were analysed with either linear mixed-effect models, mixed effect models under inequality or ordinal logistic regressions (Table 1). All statistical analysis were preformed using R version 4.3.2 [73].

**Table 1:** Outcome variables measured and associated analysis.

Variable	Measure	Delivery	Analysis
Impression	General impression	Post-exposure	Marginal model for correlated ordinal multinomial responses
	Colour	Post-exposure	Marginal model for correlated ordinal multinomial responses
	Intensity	Post-exposure	Marginal model for correlated ordinal multinomial responses
	Disturbances	Post-exposure	Marginal model for correlated ordinal multinomial responses
	Open-ended question	Post-exposure	Content analysis (descriptive)
Feeling of safety	Adapted version of the perceived personal danger scale	Post-exposure	Linear mixed effect model under inequality constraints
	Prospect	Post-exposure	Marginal model for correlated ordinal multinomial responses
	Escape	Post-exposure	Marginal model for correlated ordinal multinomial responses
	Perceived impact of light on accidents	Post-exposure	Linear mixed effect model under inequality constraints
Well-being	Positive Affect Schedule (PANAS)	Pre- and post-exposure	Linear mixed-effect model
	Negative Affect Schedule (PANAS)	Pre- and post-exposure	Linear mixed effect model under inequality constraints
	Self-reported stress	Pre- and post-exposure	Marginal model for correlated ordinal multinomial responses
	Salivary cortisol	Pre- and post-exposure	Linear mixed-effect model

We used linear mixed-effect models, ran with R package lme4 [74], for all continuous outcome variables, namely the perceived personal danger scale, the perception of the risk of accidents, the positive and negative PANAS and the cortisol responses. Linear mixed model-effect models are recommended to explicitly model the structure of hierarchical data, in which correlations of responses can be expected based on association between the participants [75]. Linear mixed-effect models assume a random distribution of residuals and homogeneity of variance (homoscedasticity) [75]. Adherence to those assumptions were checked with the R package performance [76]. Multiple comparisons for the significant model (cortisol) were then explored with the Tukey post hoc test, carried out with the R package multcomp [77]. For the outcomes whose models did not fulfil the mixed-effect models assumptions, namely the perceived personal danger scale, the perception of risks of accidents and the negative affect, we used mixed-effect models under inequality constraints, ran through the R package CLME [78]. Those are models conducting likelihood ratio type test that are robust to non-normality and heteroscedasticity [78].

We used marginal models for correlated ordinal multinomial responses, ran with the R package multgee [79], for all rating data, namely the general impression, the impression of light intensity, colour and disturbances, the prospect, the escape, and the self-reported stress. Marginal models for correlated ordinal multinomial responses are extensions of ordered logistic regression, recommended for the analysis of rating data [80], that account for the hierarchical structure of the data by including explicit correlation of variables (similarly to the previously described random effects) [79]. These models assume proportional odds, namely that the effect is similar across thresholds. Adherence to this assumption was confirmed through nominal test of the R package ordinal [81]. Assumption of proportional odds was violated in the model for light intensity, by the inclusion of the “vision” confounding variable, and in the model for prospect, by the inclusion of the “age” confounding variable. Those variables were thus excluded in those models.

All models included the CCT as fixed effect and the session (i.e., date and time) participants were in as the random effect. Confounding variables included gender, age group, street, weather and vision. Correlations between variables were explored through the variance influence factor [82], calculated with the vif function of the car R package [83], but no variable needed to be removed due to their high correlations. No interaction was considered. For models with a pre-/post-exposure design (PANAS, cortisol, self-reported stress), we followed best-practice recommendations for longitudinal study designs [84] and used pre-exposure values as a fixed effect.

### 2.8. Descriptive analysis

The answers given to the open-ended questions were classified based on which characteristics of the light they relate to, namely the general light condition, light intensity, light colour, disturbances. Each argument was then codified as either positive or negative, and the number of positive and negative comment per theme was counted. Responses from each participant could be counted within several categories when the answer consisted of elements relating to several of the characteristics of the light. Answers to the open-ended question were not analysed statistically.

## 3. Results

### 3.1. Sample characterisation

**Fig 5:**
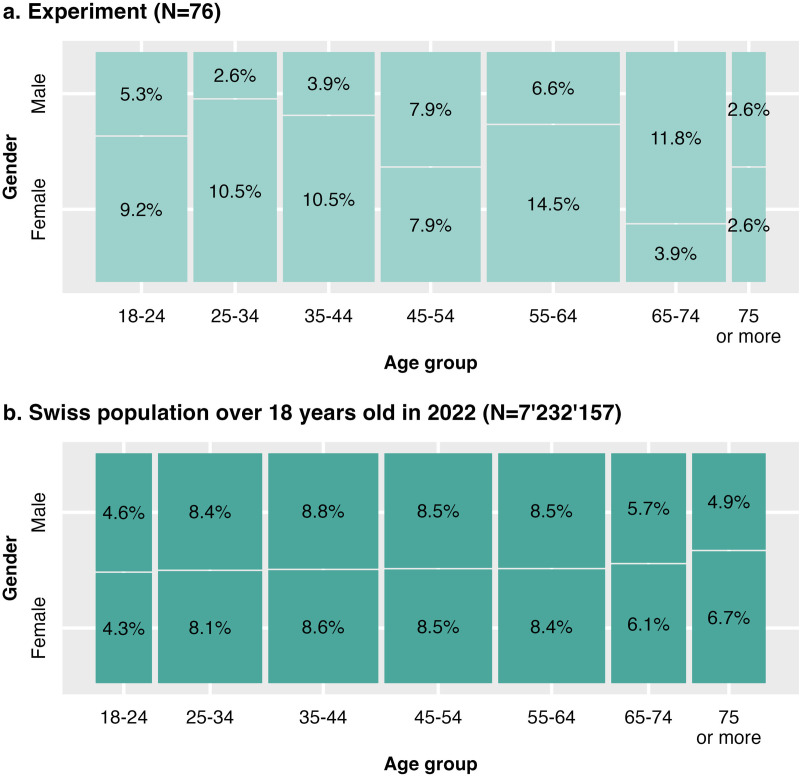
Population distribution. **(a)** Sampled population, as compared to **(b)** Swiss population, based on the Federal Statistical Office [85].

Seventy-six people where included in the analysis of the experiment, including 45 women (59.2%) and 31 men (40.8%; Table B in S1 File), distributed equally between each treatment (n = 25 for 2700K and 6500K; n = 26 for 4000K). Compared to the Swiss population, the older group category (75 years old or more) was strongly under-represented while the 18–24, 55–64, 65–74 years old were slightly over-represented (Fig 5). Most participants (47/76, 61.8%) were recruited through the flyers and thus lived in Richterswil, yet there were also some participants from other Swiss regions. Out of those 76 respondents, 62 provided answers to the open-ended question. Of those, 21 were exposed to 2700K, 19 to 4000K and 23 to 6500K.

### 3.2. Impression of light characteristics

**Fig 6:**
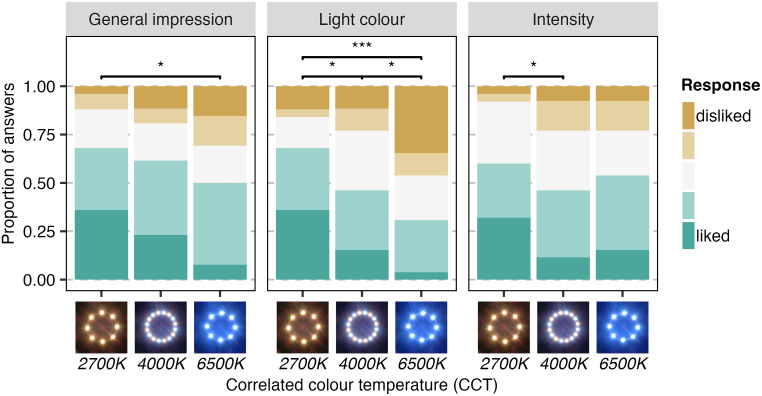
Significant differences in the impression of the light settings in terms of the general impression, the colour temperature and the intensity. Significance levels are indicated by *p < 0.05, **p < 0.01, ***p < 0.001.

The impression of the general light situation, of the CCT and of the light intensity varied based on participants’ exposure to different CCT. While evaluating the general light situation, hence answers to “do you like the lighting of this street”, responses varied from “not at all” to “extremely”, with the median answer being “quite a bit” (Table C in S1 File). Participants ranked the general impression of the light, hence answers to “do you like the lighting of this street”, of the 2700K streetlamps higher than the 6500K (est = 1.268, SE = 0.527, z = 2.408, p = 0.016*, 95%CI 0.236–2.3; Fig 6; Table D in S1 File), but no other differences between CCT were found (2700K-4000K: est = 0.646, SE = 0.609, z = 1.061, p = 0.289, CI −0.548–1.84). The only confounding variable with an impact on the general impression of light was the street, with Dorfstrasse Süd being ranked higher than Dorfstrasse Nord (est = 1.187, SE = 0.594, z = 1.997, p = 0.46*, 95%CI 0.022–2.351). Reflecting the differences in ranking the CCT, out of 12 participants describing their general impression of 6500K streetlamps in the open-ended question, eight (67%) described them negatively, using expressions such as unnatural, unfriendly, uncomfortable and tiring (Table 2). Conversely, out of the 11 participants describing their general impression of the 2700K streetlamps, eight (73%) described them positively, using expressions such as friendly, comfortable and leading to a cosy atmosphere.

**Table 2:** Expressions used to describe artificial light. Number of responses add up to more than the total number of respondents as some used several expressions.

	Correlated colour temperature	Positive expressions	Negative expression
General impression	2700K	Beautiful reflection on the floor (n = 1) Comfortable (n = 4) Even illumination (n = 2) Friendly (n = 1) Good distance between the lamps (n = 2) Not flashing (n = 1) One sees enough to feel good (n = 1) Well-lit (n = 2)	Blinding (n = 4) Disturbing (n = 1)
	4000K	Nice lamp form (n = 2) Beautiful (n = 2) Christmassy (n = 1) Comfortable (n = 1) Easy eye-adjustment to darkness (n = 1) Well-lit (n = 1) Good distance between the lamps (n = 1)	Blinding (n = 7) Gives headaches (n = 2) Not enough lamps (n = 1) Too many lamps (n = 1) Not appealing, inviting or cosy (n = 1) Not subtle (n = 1) Strangely modern (n = 1) Uncomfortable (n = 1)
	6500K	Calming (n = 2) Acceptable (n = 1) Nice lamp form (n = 1) Well-lit (n = 1)	Blinding (n = 2) Tiring (n = 1) Too many lamps (n = 1) Uncomfortable (n = 1) Unfriendly (n = 1) Unnatural (n = 1) Prefer the one down the street (4000K; n = 1)
Light colour	2700K	Warm (n = 3) Comfortable (n = 1) Familiar (n = 1)	Irregular (n = 1) Too white (n = 1)
	4000K	Comfortable (n = 2) Colourful (n = 1)	Cold or too cold (n = 4; 1) Needs more warm light, less white (n = 1) Repellent (n = 1)
	6500K		Cold or too cold (n = 4; 5) Too blue (n = 1) Too white (n = 1) Cold like a stadium floodlights (n = 1)
Light intensity	2700K	Not too bright (n = 1)	Too bright (n = 5) Side streets very dark (n = 2) Almost too dark, leading to a blinding effect with car lights (n = 1)
	4000K	Not too bright (n = 1) Comfortably bright (n = 1)	Too bright (n = 6) Very bright (n = 1) Could be dimmed after midnight in summer (n = 1) Hard to ignore (n = 1)
	6500K	Bright (n = 3) Not too bright (n = 2) One sees more when it is bright like this (n = 1)	Too bright (n = 6) Very bright (n = 2)
Disturbances	2700K	Car lights (n = 3) Store front lights (n = 2)	
	4000K		
	6500K	Car lights (n = 2) Store front lights (n = 1)	

Focusing only on the impression of the light colour when being asked “do you like the colour of the light in this street?”, responses varied from “not at all” to “extremely”, with the median answer being “moderately” (Table C in S1 File). Participants ranked the 2700K light setting higher than either the 4000K (est = 0.951, *SE* = 0.405, *z* = 2.346, *p = *0.019*, 95%CI 0.157–1.756; Fig 6; Table E in S1 File) or the 6500K (est = 1.978, *SE* = 0.577, *z* = 3.429, *p = *0.001***, 95%CI 0.847–3.109), and the 4000K higher to the 6500K (est = 1.027, *SE* = 0.411, *z* = 2.498, *p = *0.012*, 95%CI 0.221–1.833). The only confounding variable with an impact on the impression of the light colour was the street, with Dorfstrasse Süd being ranked higher than Dorfstrasse Nord (est = 1.241, SE = 0.454, *z* = 2.733, *p = *0.006**, 95%CI 0.351–2.131). Out of seven participants describing the 2700K light colour in the open-ended question, four described it positively, describing it as warm, familiar and comfortable, whereas seven out of 9 (78%) described the 4000K light colour negatively and all 12 comments on the 6500K light colour were negative, describing it as too white and cold.

Impression of the light intensity, in answer to the question “do you like the intensity of the light in this street?”, varied from “not at all” to “extremely”, with the median answer being “quite a bit” (Table C in S1 File). Light intensity of the 2700K lights was ranked higher than that of the 4000K (est = 0.981, *SE* = 0.466, *z* = 2.108, *p = *0.035*, 95%CI 0.069–1.894; Table F in S1 File). The only confounding variable with an impact on the impression of the light intensity was the street, with Dorfstrasse Süd being ranked higher than Dorfstrasse Nord (est = 1.355, *SE* = 0.319, *z* = 4.246, *p* < 0.001***, 95%CI 0.73–1.981). Responses to the open-ended question about light intensity were mostly negative (27/36 comments, 75%), with most positive comments (6/9, 66.7%) expressed by participants exposed to 6500K. Negative comments described the light as either too bright and blinding, especially when looked at directly or as *“almost too dark”*.

Perception of the disturbances caused by the light, in answer to the question “Does the lighting of this street bother you in any way (e.g., glare, headache)?”, varied from “not at all” to “extremely”, with the median answer being “a little” (Table C in S1 File). There were no significant differences in how disturbing the participants found the different light colours (2700K-4000K: est = −0.772; *SE = *0.438, *z = *−1.761*, p* = 0.078, 95%CI = −1.63–0.087; 2700K-6500K: est = −0.753, *SE = *0.638, *z* = −1.180, *p* = 0.238, 95%CI −2.004-0.498; Table G in S1 File). The only confounding variable with an impact was the street, with Dorfstrasse Süd being ranked lower than Dorfstrasse Nord (est = −1.488; *SE = *0.511, *z = *−2.911*, p* = 0.0004**, 95%CI = −2.49- −0.486). Four participants mentioned other disturbances linked to the light situation in the open-ended question. Of those three (75%) were related to the blinding effect of cars and one of storefronts. Three of those four (75%) negative comments were expressed by participants exposed to 2700K lights.

### 3.3. Psychological indicators of safety and well-being

Participants scored relatively high on the perceived personal danger scale, with an average (+/-SD) of 10.961 (+/- 4.718), out of a possible score ranging from −15–15 (Table H in S1 File). The perceived personal danger did not vary based on exposure to different CCT (2700K-4000K: est = 0.712, *t* = 0.614, *p* = 0.331; 2700K-6500K: est = 1.299, *t* = 1.083, *p* = 0.186; Table I in S1 File). No confounding variable had an impact on the ratings of the perceived personal danger. Five respondents gave comments in the open-ended question relating on safety, including two exposed to 2700K lights, one to 4000K and two to 6500K lights (Table 2). Out of those five comments, three (60%, one from each CCT) described a high feeling of safety, stating how *“one sees enough to feel […] safe”* or *“one sees more when it’s bright like now, and one feels safer”*. The other comments included one neutral and one negative, which only stated *“unsafe”*. Similarly, perceptions of the prospect and escape opportunities in the street were also relatively high (median = 2, min = −3, max = 3 for both; Table J in S1 File). The perception of the prospect did not change according to CCT (2700K-4000K: est = 0.057, *SE* = 0.401, *z* = 0.143, *p* = 0.886, 95%CI −0.728–0.846; 2700K-6500K: est = 0.447, *SE* = 0.434, *z* = 1.029, *p* = 0.304, 95%CI −0.405–1.298; Table K in S1 File), nor did the perception of the escape opportunities (2700K-4000K: est = −0.350, *SE* = 0.481, *z* = −0.728, *p* = 0.467, 95%CI −1.292-0.592; 2700K-6500K: est = −0.109, *SE* = 0.452, *z* = −0.241, *p* = 0.809, 95%CI −0.994–0.777; Table L in S1 File). Two confounding variables had an impact on prosect, namely gender with females perceiving a lower prospect (est = −0.756, *SE* = 0.373, *z* = −2.024, *p* = 0.043*, 95%CI −1.488- −0.024) and vision, with the participants wearing glasses having a lower prospect (est = −0.746, *SE* = 0.357, *z* = −2.089, *p* = 0.037*, 95%CI −1.446- −0.046). No confounding variable had an impact on the perception of escape opportunities. Participants perceived the light to have only a low impact on accidents, scoring them on average (+/-SD) of 3.137 (+/-2.07) on a scale ranging from −3–3 (Table M in S1 Supporting Information). They was no differences of the perceived influences of light on the number of accidents based on CCT (2700K-4000K: est = 0.7, *t* = 1.246, *p* = 0.155; 2700K-6500: est = 0, *t* = 0, *p* = 1; Table N in S1 Supporting Information). The only confounding variable with an impact on the perceived risk of accident was the age, with the perceived risk of accidents decreasing with age (est = −0.959, *SE* = 0.477, 95%CI −1.894- −0.025).

**Fig 7:**
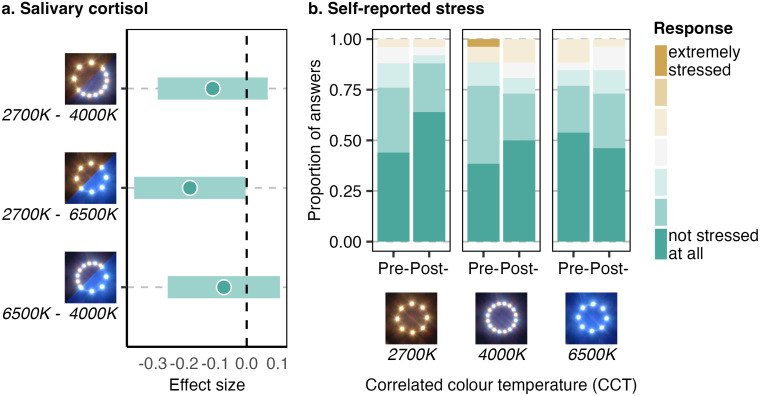
Stress responses to different light colours. **(a)** Significant decrease in salivary cortisol after exposure to 6500K lights as opposed to 2700K. **(b)** This decrease in the primary stress hormone is not perceived by the participants.

Positive affect was at an average (+/-SD) of 2.939 (+/-0.725) and negative affect of 1.152 (+/-0.220), on scales ranging from −2–2 (Table O in S1 File). Exposure to different CCT was associated neither with positive affect (2700K-4000K: est = −0.050, *SE* = 0.132 *t* = −0.377, p = 707, 95% CI −0.318–0.214; 2700K-6500K: est = 0.021 *SE* = 0.134, *t* = 0.155, p = 0.877, 95% CI −0.248–0.287; Table P in S1 File) nor with negative affect (2700K-4000K: est = 0.060, *t* = 1.184, *p* = 0.162; 2700K-6500K: est = 0.085, *t* = 1.610, *p* = 0.079; Table Q in S1 File). No confounding variable had an impact on the positive affect, while the only confounding variable with an impact on the negative affect was the street, with Dorfstrasse Süd being ranked higher than Dorfstrasse Nord (est = 0.106, *SE* = 0.053, 95%CI 0.002–0.211). Self-reports of stress levels were also very low, ranging from −3 (“not stressed at all”) to 1 (3 being “totally stressed”), with a median of −3 (Table R in S1 File). They were equally stable across CCT (2700-4000K: est = −0.612, *SE* = 0.525, *z* = −1.166, *p = *0.244, 95%CI −1.641-0.417; 2700K-6500K: est = −0.768, *SE* = 0.640, *z* = −1.199, *p = *0.230, 95%CI −2.023–0.487; Fig 7b; Table S in S1 File). The only confounding variable with an impact on the self-reported stress is the pre-exposure level, with a negative correlation (est = −0.729, *SE* = 0.144, *z* = −5.06, *p* < 0.001***, 95%CI −1.011- −0.447).

### 3.4. Physiological responses

Average (+/-SD) concentrations of cortisol pre-exposure of 1.482 nmol/l (+/-1.387) while they significantly decreased to 1.214nmol/l (+/-1.214) after exposure (est = 0.570, *SE* = 0.023, *t* = 24.822, *p* < 0.001***, 95%CI 0.525–0.616, Tables T-U in S1 File). Hormonal measurements of stress varied with exposure to different CCT. After exposure, participants under 6500K lights had lower cortisol concentrations that those exposed to 2700K (est = −0.182; *SE* = 0.076, *t* = −2.388; *p = *0.020*, 95%CI −0.335- −0.026; Fig 7a, Table U in S1 File). There were no significant differences in cortisol levels between exposure at 2700K and 4000K (est = −0.109, *SE* = 0.076, *t* = −1.448, *p* = 0.152, 95%CI −0.259–0.041) nor between 4000K and 6500K (est = 0.073, *SE* = 0.077, *t* = −0.953*, p* = 0.341*,* 95%CI −0.253–0.107). In addition to pre-exposure, two confounding variables had an impact on salivary cortisol, namely age, with an increase of cortisol with age (est = 0.005, *SE = *0.002, t = 2.555, p = 0.013*, 95%CI 0.001–0.009) and the street, with participants in Dorfstrasse Süd having lower cortisol levels than in Dorfstrasse Nord (est = 0.165, *SE = *0.080, *t = *2.078, *p = *0.041*, 95%CI −0.004–0.329).

## 4. Discussion

In a small Central European city setting, warm streetlight CCT were preferred despite larger decreases in hormonal stress levels taking place in cool white colour temperatures. There was no impact of CCT on either the feeling of safety or the affect of the participants. Consequently, we confirmed our hypothesis that perception of light would differ according to CCT, with the warm light more likely to be favoured. We however refuted our hypothesis that there would be differences in affect, feeling of safety and perceived stress based on CCT, and the difference in hormonal stress occurred in the opposite direction as predicted. Consequently, our unique study design combining real-life environment controlling the street characteristics with changes in public light CCT offers insightful perspectives into well-being’s impact of public lighting in typical small towns and allows for a better integration of residents’ views into decision-making.

### 4.1. The role of CCT for safety

Safety is the most used argument for installing numerous and bright streetlamps, and lighting one of the key environmental factors influencing perception of safety at night [86]. Here, we confirm previous studies showing no differences in feeling of safety in urban environment in terms of general feeling of safety [32,38]. We further detail those by showing this lack of impact to be consistent across three facets of safety, namely the perceived personal danger, the perceived impact on accident, and the prospect and escape. However, we also acknowledge that such lack of relationship might be drastically different in environments considered less safe. For instance, focusing on neighbourhoods considered dangerous, Painter [44] reports a decrease in both incidents of crime and disorder and fear of crime following an improvement of street lighting from yellow low pressure sodium to amber HPS, confounded by increased light intensity. Similarly, US students in peri-urban forested areas experienced lower feeling of safety if the environment was lit with amber rather than warm lights [47]. These differences hint to an impact of CCT on the feeling of safety in areas perceived less safe.

There is a recognised link association between light intensity on the feeling of safety [24], and an interaction with CCT is not to be dismissed. We here confirm that the perception of light intensity is influenced by CCT [48]. This supports the interplay found by Painter [44] between those two light characteristics and emphasise the need to consider both simultaneously in further studies. By highlighting the lack of impact of light colour temperature on the feeling of safety in a safe environment, we highlight the need for context-dependent approaches to manage the feeling of safety, and hint toward a relationship with light intensity which means that, in safe environments, other measures than adjustments in CCT might be more efficient to improve the feeling of safety.

### 4.2. The role of CCT for stress

One of our surprising results was the larger decreases in cortisol levels in cool white lights. There are to our knowledge no other studies measuring physiological stress with either anthropogenic light intensity or CCT in outdoor public spaces. Here, we found salivary cortisol level consistent with what would be expected with an assumed waking up time of 7am and a decrease over the exposition time as expected within the normal fluctuations of the circadian rhythm [87]. This decrease was however stronger under cool white lights than under warm lights. One potential explanation would be related to the Attention Restoration Theory. Previous studies on anthropogenic light, based on photo stimuli, showed more restoration and hence lower stress levels when the lamps served to highlight natural features of the environment [52]. The features highlighted by the streetlamps in our experiments were not natural and did not vary according to the CCT. However, daylight colour temperatures are always above 3750K, and whiter in clear weather [53]. The physiological responses induced by the cool white lights in our study might reflect a CCT which would happen in a safer daytime environment, with good visibility. This explanation would be supported by the studies in indoor environments, showing higher positive affect in whiter lights [26,27]. A second hypothesis as to why the decrease in stress level was larger in cool white light relates to light intensity. Our light intensity levels were consistent across CCT, yet white and cool white light can be perceived as more bright than warm light [48]. Similarly, though not asked directly about intensity levels, people were more likely to dislike the intensity of the white light as opposed to the warm light. Yet the relationship between light intensity and well-being is more agreed upon. High light intensity, by increasing the feeling of safety, tend to decrease stress, up to a plateau [88]. Consequently, a perceived higher light intensity could lead, as seen here, to lower cortisol levels.

Despite the impact of CCT on hormonal stress, there were no difference in self-reported stress. A trend was however likely in the self-reported stress, meaning that longer exposition or higher participant numbers might have led to an alignment of the hormonal and self-reported stress. However, other factors such as the attention or the fatigue might affect the cortisol levels while not being detected with self-reports. Additionally, cortisol reactions to light likely follow a nonvisual pathway [89]. Humans are not able to identify accurately the season or time of day based on natural light [90]. Consequently, a physiological and nonvisual reaction could likely differ from a more conscious self-report.

### 4.3. The role of CCT for well-being

Perceptions and preferences have long been described as expressions of our underlying unconscious needs for well-being [29]. Our clear results, confirming our hypothesis, show a higher appreciation of warmer colours might thus reflect some undetected well-being impacts. This hints that warm lights might be the comfortable and restorative environment required for well-being [29,52]. Wearing glasses or contact lenses did not change these perceptions, despite former studies showing that respondents with glasses find streetlamps brighter and more comfortable that those not wearing any [38]. Although there is no consensus on favoured CCT, this preference for warm lights has already been identified in many studies within outdoor urban settings [32,33,34]. For instance, some studies show that warm lights are perceived as more comfortable [34,40], which is a term often used by our respondents. Several factors could explain those perceptions. Firstly, there might be a habituation mechanism. In Europe, only about 10% of streetlamps are currently LED [91]. In the Zürich region of Switzerland, where this study was conducted, previously installed lights were warm white. This means that the adult population experienced, for the largest part of their life, a warm night-time illumination, and several preferences studies on environmental characteristics, including anthropogenic light [92], highlight a preference for the status-quo. Secondly, though the nonvisual pathways may induce a hormonal response linked to daylight-like situations, participants were consciously aware of the night-time environment. This might have influenced perceptions, leading people to reject daylight-like CCT, usually above 3750K [53]. Light colours representing the transition between night and day, around the 2800K CCT, are thus more likely to be perceived as natural and desirable.

Conversely, the affect, our other measure of well-being, did not differ based on CCT. There might be several explanations explaining why we did not detect an impact. Firstly, and similarly to the self-reported stress, it might be due to the short duration of the exposure. Even though the PANAS can be recommended for short-term variations [93], the similarity between the settings might lead to slower changes in affect. However, most pedestrians use the street for an average of 15 min [64], meaning that a significantly longer exposure would decrease the relevance of the results. Secondly, the PANAS scale has also been criticised for its failure to detect some affect, such as happiness, calmness and content [94]. Tiredness, for instance, has been shown to respond to light colour [25] and is not included in the PANAS. Though the PANAS includes alertness, known to be influenced by light colour [26–28], a restorative aspect leading to a calmer state might be less likely to be detected.

The mismatch between the perceptions, and well-being, with the physiological responses could also be explained by the relaxed state during which participants found themselves during the experiment. Although public spaces are usually used actively [64], our participants were sitting down during the experiment and did not to move or orient themselves. Their perceived comfort might thus have played a stronger role in their ratings. Indeed, previous studies show that despite the comfort provided by the warm colours, whiter tones were rated better in term of their quality, including their influence on the ability to orient oneself [34,40]. The well-being benefits of light colour depending on the active and passive use of public spaces might thus need to be further explored. Irrespectively of the reasons for behind those perceptions and affect non-responses, including residents’ perceptions in urban planning can improve the acceptability of measures, which is critical for their implementation, and help set priorities for urban sustainability.

### 4.4. Anthropogenic lighting in the context of urban sustainability

Well-being is a key aspect of urban lightning, yet far from the only aspect to consider for sustainability. Anthropogenic light also strongly influences energy consumption and biodiversity. There is thus a need to define priorities in light planning. White LED are often prioritised due to their lower energy use, and thus costs, and we show here that they have a stronger impact on decreasing stress levels. Yet given that fear of lack of support from the residents hinders municipalities’ implementation of light pollution reduction measures [95], following preferences for a comfortable night-time environment, with warm LEDs, might be more judicious, in particular as this would allow synergies to be used for urban sustainability. For instance, the warm lights favoured by residents are increasingly recognised to have lower impact on biodiversity: warmer light colour temperature are less detrimental to night-flying insects [10–12,96], though there seem to be little impact on either bats or birds [10,13]. And whereas residents might not recognise the importance of CCT for biodiversity, there is a strong understanding within the Swiss population that anthropogenic light at night is harmful for night-active animals and a similar wish to act against it [92]. Consequently, highlighting such synergies with biodiversity conservation might contribute to increased support for CCT adjustments. A similar argument could be made about energy consumption. Energy consumption also plays a big role in the sustainability debate [97] yet including LEDs, irrespectively of their CCT, instead of the current HPS streetlamps already provides high potential for energy saving [7].

### 4.5. Strengths, limitations and ways forward

This is one of the first field studies to use a parallel group design with controlled environment for different light colours. We also combined self-reports with physiological measurements, which allows for a more fine-tuned understanding of responses. Nevertheless, some limitations must be considered. First, our study has a relatively low number of participants, though within the same range of many other studies on anthropogenic light [38–40,46,88] and relatively well aligned to the Swiss population in terms of age and gender. Field research in general often rely on smaller sample sizes than laboratory studies, leading to weaker effects, yet might represent “real life” situations better. Second, environmental or architectural conditions such as air temperature or building reflections could not be included in the analysis and might need to be captured better in other studies. In particular, our study site mostly consisted of old buildings on which light is likely to reflect itself very different than on new builds. Third, light quality could also be influenced by minute characteristics linked to LED array brands, which would limit the generalisation of our results. Fourth, although we included the use of glasses or contact lenses in our analysis, we did not go into details about any eye conditions that could affect the perception of light. This was done on purpose for anonymity purposes, yet conditions such as myopia could have a stronger impact on some of our outcome variables, such as the prospect which particularly relies on long-distance vision [45]. However, previous research focusing on people with visual impairments found results consistent with most of the literature on preferences for CCT [34]. Fifth, the use of public spaces in mostly done in a dynamic manner, while walking [64], while our study design, compelled by technical possibilities, forced a static use of public space. Investigating well-being impacts of different light conditions during walks could provide results that better align with real-life conditions. Sixth, we cannot exclude a self-selection bias whereas participants who volunteered might have preconceived ideas about ideal lighting situations. Seventh, we acknowledge that our results are only representative of relatively safe small European towns, our study site being a 20 km/h zone with many small shops and a low crime rate. Perceptions of safety and urban planning are however culturally dependant, with large variations world-wide, and residents of other world regions might have extremely different responses to light colour. We thus highlight the need to not only study the impact of light in the Global North, but also in regions of fast-growing urban centres where proactively planning sustainable public lightning might help avoid the light pollution issues rather than having to retroactively mitigate them.

## 5. Conclusions

Well-being responses to artificial lights were diverse. Physiological responses showed the a stronger decrease in stress hormones in cool white lights, while warm lights are preferred. The discrepancy between this hormonal response and the preferences could be explained by a mismatch of expectations: whereas respondents physiologically responded to daytime situations in cool white light, their self-reports adjusted to the night-time environment and habit. Yet this mismatch highlights the trade-offs needed while planning urban public lighting.

In sum, higher appreciation of warmer light colour highlight useful synergies allowing to mitigate public lightings’ impact on biodiversity and health, and strategies to put people’s preferences in the centre when either mitigating or decreasing the energy consumption of public lighting, yet at the expense of stress levels. Arguments for defining priorities on light adjustments should rely on potential synergies with other facets of sustainability to gather more support and emphasize win-win situations.

## Supporting information

S1 File (1) Pre- and (2) post-exposure questionnaires; (3) Study design, including weather conditions and number of participants; (4) Output and model summaries for each variables.
(PDF)
