# Supplementary material for: Randomised trial reveals a mismatch between preferences for and hormonal responses to anthropogenic light colour temperatures
Source: PLoS One. 2025 Aug 11;20(8):e0327843. doi: 10.1371/journal.pone.0327843 (PMC12338827; doi:10.1371/journal.pone.0327843)
Supplement: S1 File — (1) Pre- and (2) post-exposure questionnaires; (3) Study design, including weather conditions and number of participants; (4) Output and model summaries for each variables. (PDF) [file pone.0327843.s001.pdf]

Supplementary Information to "Randomised trial reveals a mismatch between preferences for and hormonal responses to anthropogenic light colour temperatures"

Solène Guenat[1]*; Jörg Haller[2]; and Nicole Bauer[1]
2025. *PLOS One*, DOI: 10.1371/journal.pone.0327843

[1] Social Sciences in Landscape Research, Economics and Social Sciences, Swiss Federal Institute for Forest, Snow and Landscape Research, Zürcherstrasse 111, 8903 Birmensdorf, Switzerland
[2] Elektrizitätswerk des Kantons Zürich, Dreikönigstrasse 18, 8022, Zürich, Switzerland
* corresponding author: solene.guenat@wsl.ch; 0041 44 739 21 01

Table of content

1. Pre-exposure questionnaire 2
2. Post-exposure questionnaire 3
3. Study design
    - Table A. Weather conditions during each experimental session 7
    - Table B. Number of participants for each explanatory variable 8
4. Results
    - Table C. Output summaries for the impression of the light 9
    - Table D. Model summary for the general impression of the light 10
    - Table E. Model summary for the impression of the light colour 11
    - Table F. Model summary for the impression of the light intensity 12
    - Table G. Model summary for the impression of the light disturbances 13
    - Table H. Output summary for the perceived personal danger scale 14
    - Table I. Model summary for the perceived personal danger scale 14
    - Table J. Output summary for the prospect-escape theory 15
    - Table K. Model summary for the prospect 16
    - Table L. Model summary for the escape 17
    - Table M. Output summary for the perceived risk of accidents 18
    - Table N. Model summary for the perceived risk of accidents 18
    - Table O. Output summary for the affect 19
    - Table P. Model summary for the positive affect 20
    - Table Q. Model summary for the negative affect 21
    - Table R. Output summary for the self-reported stress 22
    - Table S. Model summary for the self-reported stress 22
    - Table T. Output summary for the salivary cortisol 23
    - Table U. Model summary for the salivary cortisol 24

1. Pre-exposure questionnaire

1.1. Well-being

First, we would like to know how you feel. The following words describe different kind of feelings and emotions. Read each word and mark the intensity on the scale. You have the choice between five levels. Please indicate how you feel right now, that is, at the present moment.

	not at all	a little	moderately	quite a bit	extremely
active	☐	☐	☐	☐	☐
distressed	☐	☐	☐	☐	☐
interested	☐	☐	☐	☐	☐
excited	☐	☐	☐	☐	☐
upset	☐	☐	☐	☐	☐
strong	☐	☐	☐	☐	☐
guilty	☐	☐	☐	☐	☐
scared	☐	☐	☐	☐	☐
hostile	☐	☐	☐	☐	☐
inspired	☐	☐	☐	☐	☐
proud	☐	☐	☐	☐	☐
irritable	☐	☐	☐	☐	☐
enthusiastic	☐	☐	☐	☐	☐
ashamed	☐	☐	☐	☐	☐
alert	☐	☐	☐	☐	☐
nervous	☐	☐	☐	☐	☐
determined	☐	☐	☐	☐	☐
attentive	☐	☐	☐	☐	☐
jittery	☐	☐	☐	☐	☐
afraid	☐	☐	☐	☐	☐

1.2. Stress

Please select how stress you feel right now.

not stressed at all ☐ ☐ ☐ ☐ ☐ ☐ ☐ *extremely stressed*

2. Post-exposure questionnaire

2.1. General impression

We would like to ask you to think about the lighting of this street and to answer the following questions.

	not at all	a little	moderately	quite a bit	extremely
Do you like the lighting of this street?	☐	☐	☐	☐	☐
Do you like the colour of light in this street?	☐	☐	☐	☐	☐
Do you like the intensity of the light in this street?	☐	☐	☐	☐	☐
Does the lighting of this street bother you in any way (e.g. glare, headache)?	☐	☐	☐	☐	☐

Do you have additional comments on the lighting of this street?

....

....

....

....

....

....

....

....

2.2. Feeling of safety

Please select how you feel right now.

not safe at all ☐ ☐ ☐ ☐ ☐ ☐ ☐ extremely safe

Thinking about this street, please indicate to which extent you agree with the following statements.

	totally disagree … totally agree
I have an unpleasant feeling at this street.	☐ ☐ ☐ ☐ ☐ ☐ ☐
I feel uneasy in this street.	☐ ☐ ☐ ☐ ☐ ☐ ☐
I think that the lighting of this street could lead to fewer accidents.	☐ ☐ ☐ ☐ ☐ ☐ ☐
I think that the lighting of this street could lead more accidents.	☐ ☐ ☐ ☐ ☐ ☐ ☐
I would not mind walking along this street unaccompanied.	☐ ☐ ☐ ☐ ☐ ☐ ☐
From this street, I have a good overview of my surroundings.	☐ ☐ ☐ ☐ ☐ ☐ ☐
I would feel safe to walk along this street at night.	☐ ☐ ☐ ☐ ☐ ☐ ☐
I would quickly get away from this street.	☐ ☐ ☐ ☐ ☐ ☐ ☐
In case of threatening danger, I could easily escape from this street.	☐ ☐ ☐ ☐ ☐ ☐ ☐
I would feel anxious if I had to walk along this street at night.	☐ ☐ ☐ ☐ ☐ ☐ ☐
I would take a long detour to avoid this street.	☐ ☐ ☐ ☐ ☐ ☐ ☐

2.3. Well-being

Now we would like to know how you feel. The following words describe different kind of feelings and emotions. Read each word and mark the intensity on the scale. You have the choice between five levels. Please indicate how you feel right now, that is, at the present moment.

	not at all	a little	moderately	quite a bit	extremely
active	☐	☐	☐	☐	☐
distressed	☐	☐	☐	☐	☐
interested	☐	☐	☐	☐	☐
excited	☐	☐	☐	☐	☐
upset	☐	☐	☐	☐	☐
strong	☐	☐	☐	☐	☐
guilty	☐	☐	☐	☐	☐
scared	☐	☐	☐	☐	☐
hostile	☐	☐	☐	☐	☐
inspired	☐	☐	☐	☐	☐
proud	☐	☐	☐	☐	☐
irritable	☐	☐	☐	☐	☐
enthusiastic	☐	☐	☐	☐	☐
ashamed	☐	☐	☐	☐	☐
alert	☐	☐	☐	☐	☐
nervous	☐	☐	☐	☐	☐
determined	☐	☐	☐	☐	☐
attentive	☐	☐	☐	☐	☐
jittery	☐	☐	☐	☐	☐
afraid	☐	☐	☐	☐	☐

2.4. Stress

Please select how stressed you feel right now.

not stressed at all ☐ ☐ ☐ ☐ ☐ ☐ ☐ *extremely stressed*

2.5. Demographic information

Do you wear glasses?

☐ Yes
☐ No
☐ Unregularly. I had them on during the study.
☐ Unregularly. I did not have them on during the study.

Do you wear contact lenses?

☐ Yes
☐ No
☐ Unregularly. I had them on during the study.
☐ Unregularly. I did not have them on during the study.

What is your gender?

☐ Female
☐ Male
☐ Other

To which age group do you belong?

☐ 18-24 years old
☐ 25-34 years old
☐ 35-44 years old
☐ 45-54 years old
☐ 55-64 years old
☐ 65-74 years old
☐ 75 years old or more

3. Study design

Table A. Weather data during each experimental session. The weather data was obtained for every 10min, from the nearby Wädenswil wetter station.

<u>Source:</u> Federal Office of Meteorology and Climatology MeteoSwiss (data.geo.admin.ch)

Date	Session	Air temperature 2m above ground (°C, average)	Precipitations (mm, total)	Wind speed (m/s, average)	Relative air humidity 2m above ground (%, average)
01.11.2023	Early	9.29	0.00	6.11	78.29
01.11.2023	Late	7.21	0.00	5.69	85.29
02.11.2023	Early	8.03	0.00	6.67	89.27
02.11.2023	Late	7.83	0.10	5.30	90.57
03.11.2023	Early	5.54	0.00	9.10	81.39
03.11.2023	Late	4.09	0.00	4.47	87.90
06.11.2023	Early	7.60	2.10	16.60	80.57
06.11.2023	Late	6.87	0.40	2.35	90.12
07.11.2023	Late	5.96	0.00	8.89	78.63
08.11.2023	Early	4.57	0.00	6.17	78.14
08.11.2023	Late	2.99	0.00	3.41	83.20
09.11.2023	Late	5.24	0.00	2.67	93.63
10.11.2023	Early	4.86	0.00	7.91	75.63
10.11.2023	Late	4.94	0.00	3.91	86.03
13.11.2023	Early	10.54	0.30	9.00	95.03
13.11.2023	Late	13.49	0.00	10.50	78.44
15.11.2023	Late	6.50	0.00	4.17	86.41
16.11.2023	Early	7.67	0.30	6.23	89.34
16.11.2023	Late	13.77	0.10	42.63	54.10
17.11.2023	Early	5.73	0.40	8.06	87.13
17.11.2023	Late	5.07	0.00	12.36	85.29
20.11.2023	Early	9.74	0.00	3.37	66.53
Average		7.16	0.17	8.44	82.77
Standard error		4.27	0.25	2.92	27.31

Table B. Number of participants for each explanatory variable.

Variable	Categories	Nb respondents (n=76)
CCT	2700K	25
	4000K	26
	6500K	25
Street	Dorfstrasse Nord	26
	Dorfstrasse Süd	23
	Poststrasse	27
Weather	Rainy	34
	Not rainly	42
Vision	Direct	35
	Indirect (glasses or contact lenses)	41
Gender	Female	45
	Male	31
	Others	0
Age group	18-24 years old	11
	25-34 years old	10
	35-44 years old	11
	45-54 years old	12
	55-64 years old	16
	65-74 years old	12
	75 years old or more	4

4. **Results**

Table C. Outcome summary for the general impression of the light

	Not at all	A little	Moderately	Quite a bit	Extremely	Min	Median	Max
**General impression**								
2700K	1	2	5	8	9	Not at all	Quite a bit	Extremely
4000K	3	2	5	10	6	Not at all	Quite a bit	Extremely
6500K	3	4	5	11	2	Not at all	Quite a bit	Extremely
Total	*7*	*8*	*15*	*29*	*17*	*Not at all*	*Quite a bit*	*Extremely*
**Light colour**								
2700K	3	1	4	8	9	Not at all	Quite a bit	Extremely
4000K	3	3	8	8	4	Not at all	Moderately	Extremely
6500K	8	3	6	7	1	Not at all	Moderately	Extremely
Total	*14*	*7*	*18*	*23*	*14*	*Not at all*	*Moderately*	*Extremely*
**Light intensity**								
2700K	1	1	8	7	8	Not at all	Quite a bit	Extremely
4000K	2	4	8	9	3	Not at all	Moderately	Extremely
6500K	1	4	6	10	4	Not at all	Quite a bit	Extremely
Total	*4*	*9*	*22*	*26*	*15*	*Not at all*	*Quite a bit*	*Extremely*
**Disturbances**								
2700K	13	6	3	1	2	Not at all	Not at all	Extremely
4000K	9	9	2	4	2	Not at all	A little	Extremely
6500K	10	7	2	4	2	Not at all	A little	Extremely
Total	*32*	*22*	*7*	*9*	*6*	*Not at all*	*A little*	*Extremely*

Values represent the number of responses per category

Table D. Model summary for the general impression of the light

(Marginal model for correlated ordinal multinomial responses)

**Summary of residuals**					
Min.	1st quartile	Median	Mean	3rd quartile	Max.
-0.440	-0.197	-0.104	-0.002	-0.041	0.968

**Coefficients:**						
	Est/Beta	San.se	95% CI	San.z	p-value	Odds ratio
beta10	-4.151	1.083	-6.274 - -2.029	-3.833	<0.001***	0.020
beta20	-3.210	0.987	-5.145 - -1.275	-3.251	0.001**	0.040
beta30	-2.118	0.927	-3.935 - -0.301	-2.285	0.022*	0.120
beta40	-0.226	0.876	-1.942 - 1.49	-0.259	0.796	ns
CCT (4000K)	0.646	0.609	-0.548 - 1.84	1.061	0.289	ns
CCT (6500K)	1.268	0.527	0.236 - 2.3	2.408	0.016*	3.550
Gender (female)	-0.013	0.447	-0.89 - 0.864	-0.029	0.977	ns
Age	0.003	0.013	-0.021 - 0.028	0.274	0.784	ns
Rain	-0.095	0.413	-0.904 - 0.714	-0.230	0.818	ns
Vision (indirect)	0.625	0.479	-0.313 - 1.564	1.306	0.191	ns
Street (Dorfstrasse Süd)	1.187	0.594	0.022 - 2.351	1.997	0.046*	3.280
Street (Poststrasse)	0.630	0.560	-0.468 - 1.728	1.125	0.261	ns

Model equation: formula = GeneralImpression ~ LightColour + Gender + Age + Rain + Street +Vision, id = Session

Significance levels are indicated by *p < 0.05, **p < 0.01, ***p < 0.001, ns=non-significant

Table E. Model summary for the impression of the light colour

(Marginal model for correlated ordinal multinomial responses)

Summary of residuals					
Min.	1st quartile	Median	Mean	3rd quartile	Max.
-0.621	-0.258	-0.120	0.000	-0.035	0.970

Coefficients:						
	Est/Beta	San.se	95% CI	San.z	p-value	Odds ratio
beta10	-3.650	0.911	-5.435 - -1.864	-4.006	<0.001***	0.030
beta20	-3.121	0.824	-4.737 - -1.505	-3.785	<0.001***	0.040
beta30	-1.886	0.720	-3.296 - -0.476	-2.621	0.009**	0.150
beta40	-0.127	0.787	-1.67 - 1.415	-0.162	0.871	ns
CCT (4000K)	0.951	0.405	0.157 - 1.746	2.346	0.019*	2.590
CCT (6500K)	1.978	0.577	0.847 - 3.109	3.429	0.001***	7.230
Gender (female)	0.124	0.452	-0.762 - 1.01	0.274	0.784	ns
Age	-0.001	0.015	-0.03 - 0.029	-0.042	0.966	ns
Rain	-0.262	0.470	-1.183 - 0.659	-0.558	0.577	ns
Vision (indirect)	0.843	0.484	-0.106 - 1.792	1.740	0.082	ns
Street (Dorfstrasse Süd)	1.241	0.454	0.351 - 2.131	2.733	0.006**	3.460
Street (Poststrasse)	0.489	0.489	-0.469 - 1.448	1.001	0.317	ns

Model equation: formula = ImpressionColour ~ LightColour + Gender + Age + Vision + Rain + Street , id = Session

Significance levels are indicated by *p < 0.05, **p < 0.01, ***p < 0.001, ns=non-significant

Table F. Model summary for the impression of the light intensity

(Marginal model for correlated ordinal multinomial responses)

Summary of residuals					
Min.	1st quartile	Median	Mean	3rd quartile	Max.
-0.411	-0.271	-0.091	-0.001	-0.024	0.959

Coefficients:						
	Est/Beta	San.se	95% CI	San.z	p-value	Odds ratio
beta10	-4.604	1.049	-6.661 - -2.547	-4.388	<0.001***	0.010
beta20	-3.251	0.909	-5.033 - -1.468	-3.575	<0.001***	0.040
beta30	-1.680	0.920	-3.482 - 0.123	-1.827	0.068	ns
beta40	0.066	0.823	-1.547 - 1.68	0.081	0.936	ns
CCT (4000K)	0.981	0.466	0.069 - 1.894	2.108	0.035*	2.670
CCT (6500K)	0.633	0.425	-0.199 - 1.466	1.491	0.136	ns
Gender (female)	-0.188	0.464	-1.098 - 0.722	-0.405	0.685	ns
Age	0.013	0.013	-0.012 - 0.039	1.012	0.311	ns
Rain	-0.363	0.331	-1.012 - 0.287	-1.094	0.274	ns
Street (Dorfstrasse Süd)	1.355	0.319	0.73 - 1.981	4.246	<0.001***	3.880
Street (Poststrasse)	0.529	0.465	-0.381 - 1.44	1.140	0.255	ns

Model equation: formula = ImpressionIntensity ~ LightColour + Gender + Age + Rain + Street , id = Session

Significance levels are indicated by *p < 0.05, **p < 0.01, ***p < 0.001, ns=non-significant

Table G. Model summary for the impression of the light disturbances

(Marginal model for correlated ordinal multinomial responses)

Summary of residuals					
Min.	1st quartile	Median	Mean	3rd quartile	Max.
-0.854	-0.257	-0.105	0.006	-0.035	0.983

Coefficients:						
	Est/Beta	San.se	95% CI	San.z	p-value	Odds ratio
beta10	1.529	1.064	-0.557 - 3.615	1.437	0.151	0.010
beta20	2.847	1.037	0.815 - 4.878	2.746	0.006**	0.040
beta30	3.349	0.915	1.554 - 5.143	3.658	<0.001***	ns
beta40	4.300	0.907	2.522 - 6.078	4.740	<0.001***	ns
CCT (4000K)	-0.772	0.438	-1.63 - 0.087	-1.761	0.078	2.670
CCT (6500K)	-0.753	0.638	-2.004 - 0.498	-1.180	0.238	ns
Gender (female)	-0.141	0.451	-1.025 - 0.742	-0.313	0.754	ns
Age	-0.016	0.015	-0.044 - 0.012	-1.101	0.271	ns
Rain	0.557	0.472	-0.368 - 1.483	1.181	0.238	ns
Vision (indirect)	-0.157	0.465	-1.069 - 0.756	-0.337	0.736	3.880
Street (Dorfstrasse Süd)	-1.488	0.511	-2.49 - -0.486	-2.911	0.004**	ns
Street (Poststrasse)	-0.785	0.655	-2.067 - 0.498	-1.199	0.231	ns

Model equation: formula = ImpressionDisturbances ~ LightColour + Gender + Age + Rain + Vision + Street , id = Session

Significance levels are indicated by *p < 0.05, **p < 0.01, ***p < 0.001, ns=non-significant

Table H. Output summary for the perceived personal danger scale

	Min	Median	Max	Mean	SD
2700K	4	12	15	11.8	3.3.651
4000K	-6	12.5	15	11.154	4.872
6500K	-7	11	15	9.92	5.438
Total	*-7*	*12*	*15*	*10.961*	*4.718*

Table I. Model summary for the perceived personal danger scale

(linear mixed-effect model under inequality constraints)

**Global test**		
	Statistic	p-value
Bootstrap LRT	1.218	0.479

**Individual tests (Williams' type tests)**			
Contrast	Estimate	Statistic	P-value
4000K – 2700K	0.712	0.614	0.331
6500K – 2700K	1.299	1.083	0.186

**Variance components**	
	SD
Session	<0.001
Residual	4.117

**Fixed effect coefficients (theta):**			
	Est/Beta	SE	95% CI
CCT (2700K)	13.052	1.976	9.179 - 16.925
CCT (4000K)	12.340	1.938	8.541 - 16.138
CCT (6500K)	11.753	1.974	7.884 - 15.621
Gender (female)	0.761	1.017	-1.232 - 2.753
Age	-0.015	0.029	-0.073 - 0.043
Rain	0.575	0.987	-1.359 - 2.509
Vision (indirect)	-0.566	1.038	-2.6 - 1.468
Street (Dorfstrasse Süd)	-1.593	1.211	-3.967 - 0.781
Street (Poststrasse)	-1.106	1.182	-3.423 - 1.211

Model based on 1000 bootstrap samples
Model equation: PDS ~ LightColour + Gender + Age + Vision + Rain + Street + (1 | Session)

Significance levels are indicated by *p < 0.05, **p < 0.01, ***p < 0.001

Table J. Output summary for the prospect escape theory

	Totally disagree (-3)	-2	-1	0	1	2	Totally agree (3)	Min	Median	Max
**Prospect**										
2700K	0	1	1	1	2	6	14	-2	3	3
4000K	1	0	1	1	1	8	14	-3	3	3
6500K	0	2	1	0	4	9	9	-2	2	3
Total	*1*	*3*	*3*	*2*	*7*	*23*	*37*	*-3*	*2*	*3*
**Escape**										
2700K	1	0	1	6	2	6	9	-3	2	3
4000K	0	0	1	4	4	7	10	-1	2	3
6500K	0	0	1	3	5	8	8	-2	2	3
Total	*1*	*1*	*5*	*10*	*11*	*21*	*27*	*-3*	*2*	*3*

Non-summary values represent the number of responses per category

Table K. Model summary for the prospect

(Marginal model for correlated ordinal multinomial responses)

Summary of residuals					
Min.	1st quartile	Median	Mean	3rd quartile	Max.
-0.367	-0.062	-0.033	-0.001	-0.014	0.994

Coefficients:						
	Est/Beta	San.se	95% CI	San.z	p-value	Odds ratio
beta10	-3.215	1.381	-5.922 - -0.508	-2.328	0.020*	0.040
beta20	-2.229	0.757	-3.713 - -0.746	-2.945	0.003**	0.110
beta30	-1.694	0.670	-3.008 - -0.38	-2.527	0.012*	0.180
beta40	-1.404	0.625	-2.629 - -0.178	-2.245	0.025	0.250
beta50	-0.678	0.595	-1.844 - 0.489	-1.138	0.255	ns
beta60	0.866	0.576	-0.262 - 1.995	1.504	0.133	ns
CCT (4000K)	0.057	0.401	-0.728 - 0.843	0.143	0.886	ns
CCT (6500K)	0.447	0.434	-0.405 - 1.298	1.029	0.304	ns
Gender (female)	-0.756	0.373	-1.488 - -0.024	-2.024	0.043*	0.470
Rain	-0.364	0.295	-0.942 - 0.214	-1.235	0.217	ns
Vision (indirect)	-0.746	0.357	-1.446 - -0.046	-2.089	0.037*	0.470
Street (Dorfstrasse Süd)	-0.681	0.470	-1.601 - 0.239	-1.450	0.147	ns
Street (Poststrasse)	0.731	0.492	-0.234 - 1.695	1.485	0.138	ns

Model equation: formula = Prospect ~ LightColour + Gender + Rain + Vision + Street , id = Session

Significance levels are indicated by *p < 0.05, **p < 0.01, ***p < 0.001, ns=non-significant

Table L. Model summary for the escape

(Marginal model for correlated ordinal multinomial responses)

Summary of residuals					
Min.	1st quartile	Median	Mean	3rd quartile	Max.
-0.280	-0.142	-0.058	0.000	-0.013	0.981

Coefficients:						
	Est/Beta	San.se	95% CI	San.z	p-value	Odds ratio
beta10	-4.423	1.233	-6.839 - -2.006	-3.587	<0.001***	0.010
beta20	-3.721	1.112	-5.901 - -1.541	-3.346	0.001***	0.020
beta30	-2.430	0.804	-4.006 - -0.854	-3.023	0.003**	0.090
beta40	-1.377	0.852	-3.046 - 0.292	-1.617	0.106	ns
beta50	-0.665	0.750	-2.134 - 0.804	-0.888	0.375	ns
beta60	0.488	0.763	-1.008 - 1.983	0.639	0.523	ns
CCT (4000K)	-0.350	0.481	-1.292 - 0.592	-0.728	0.467	ns
CCT (6500K)	-0.109	0.452	-0.994 - 0.777	-0.241	0.809	ns
Gender (female)	-0.224	0.356	-0.923 - 0.475	-0.629	0.530	ns
Age	0.000	0.011	-0.022 - 0.022	0.005	0.996	ns
Rain	0.239	0.346	-0.439 - 0.918	0.691	0.490	ns
Vision (indirect)	0.171	0.450	-0.711 - 1.053	0.381	0.703	ns
Street (Dorfstrasse Süd)	0.116	0.400	-0.668 - 0.9	0.290	0.772	ns
Street (Poststrasse)	0.455	0.461	-0.449 - 1.358	0.986	0.324	ns

Model equation: formula = Escape ~ LightColour + Gender + Age +Rain + Vision + Street , id = Session

Significance levels are indicated by *p < 0.05, **p < 0.01, ***p < 0.001, ns=non-significant

Table M. Output summary for the perceived risk of accidents

	Min	Median	Max	Mean	SD
2700K	0	3.5	6	3.333	1.903
4000K	-3	3	5	2.680	1.930
6500K	-3	4.5	6	3.417	2.358
Total	*-3*	*3*	*6*	*3.137*	*2.070*

Table N. Model summary for the perceived risk of accidents

(linear mixed-effect model under inequality constraints)

**Global test**		
	Statistic	p-value
Bootstrap LRT	0.105	0.335

**Individual tests (Williams' type tests)**			
Contrast	Estimate	Statistic	P-value
4000K – 2700K	0.700	1.246	0.155
6500K – 2700K	0.000	0.000	1.000

**Variance components**	
	SD
Session	<0.001
Residual	0.957

**Fixed effect coefficients (theta):**			
	Est/Beta	SE	95% CI
CCT (2700K)	3.970	0.948	2.111 - 5.828
CCT (4000K)	3.270	0.930	1.447 - 5.092
CCT (6500K)	3.970	0.940	2.127 - 5.813
Pre-exposure	-0.682	0.492	-1.647 - 0.283
Gender (female)	0.002	0.014	-0.025 - 0.029
Age	-0.959	0.477	-1.894 - -0.025
Rain	0.132	0.500	-0.848 - 1.111
Vision (indirect)	0.129	0.576	-1 - 1.258
Street (Dorfstrasse Süd)	0.165	0.577	-0.967 - 1.297

Model based on 1000 bootstrap samples
Model equation: PercAccidents ~ LightColour + Gender + Age + Vision + Rain + Street + (1 | Session)

Significance levels are indicated by *p < 0.05, **p < 0.01, ***p < 0.001

Table O. Model summary for the affect

	Min	Median	Max	Mean	SD
**Positive**					
2700K	1.8	2.9	4.8	3.004	0.821
4000K	1.1	2.9	4.1	2.919	0.688
6500K	1.7	2.9	4.4	2.896	0.685
Total	*1.1*	*2.9*	*4.8*	*2.939*	*0.725*
**Negative**					
2700K	1	1	1.5	1.096	0.134
4000K	1	1	2.1	1.169	0.269
6500K	1	1.15	1.7	1.192	0.230
Total	*1*	*1*	*2.1*	*1.152*	*0.220*

Table P. Model summary for the positive affect

(linear mixed-effect model)

Scaled residuals				
Min.	1st quartile	Median	3rd quartile	Max
-2.348	-0.786	-0.034	0.740	2.097

Fixed Effects					
	Est/Beta	SE	95% CI	t	p
Intercept	-0.153	0.421	-0.991 - 0.688	-0.363	0.718
Pre-exposure	0.997	0.106	0.782 - 1.208	9.400	<0.001***
CCT (4000K)	-0.050	0.132	-0.318 - 0.214	-0.377	0.707
CCT (6500K)	0.021	0.134	-0.248 - 0.287	0.155	0.877
Gender (female)	-0.117	0.113	-0.341 - 0.109	-1.037	0.304
Age	0.001	0.003	-0.006 - 0.008	0.282	0.779
Rain	-0.014	0.139	-0.315 - 0.267	-0.102	0.920
Vision (indirect)	0.019	0.115	-0.209 - 0.247	0.162	0.872
Street (Dorfstrasse Süd)	-0.163	0.137	-0.435 - 0.11	-1.193	0.237
Street (Poststrasse)	-0.091	0.134	-0.359 - 0.175	-0.679	0.500

Random Effects		
	Variance	S.D.
Session (Intercept)	0.035	0.186
Residual	0.202	0.449

Model fit		
	Marginal	Conditional
R^2	0.623	0.558

P-values for fixed effects calculated using Satterthwaites approximations.
Model equation: PositivePANAS_T2 ~ PositivePANAS_T1 + LightColour + Gender + Age + Vision + Rain + Street + (1 | Session)

Significance levels are indicated by *p < 0.05, **p < 0.01, ***p < 0.001

Table Q. Model summary for the negative affect

(linear mixed-effect model under inequality constraints)

Global test		
	Statistic	p-value
Bootstrap LRT	0	0.176

Individual tests (Williams' type tests)			
Contrast	Estimate	Statistic	P-value
4000K – 2700K	0.060	1.184	0.180
6500K – 2700K	0.085	1.610	0.071

Variance components	
	SD
Session	<0.001
Residual	0.179

Fixed effect coefficients (theta):			
	Est/Beta	SE	95% CI
CCT (2700K)	0.732	0.129	0.48 - 0.984
CCT (4000K)	0.792	0.127	0.544 - 1.04
CCT (6500K)	0.817	0.133	0.556 - 1.077
Pre-exposure	0.257	0.069	0.122 - 0.392
Gender (female)	0.063	0.044	-0.024 - 0.15
Age	-0.001	0.001	-0.003 - 0.002
Rain	0.009	0.043	-0.075 - 0.093
Vision (indirect)	0.042	0.046	-0.048 - 0.132
Street (Dorfstrasse Süd)	0.106	0.053	0.002 - 0.211
Street (Poststrasse)	-0.011	0.051	-0.112 - 0.09

Model based on 1000 bootstrap samples
Model equation: NegativePANAS_T2 ~ NegativePANAS_T1 + LightColour + Gender + Age + Vision + Rain + Street + (1 | Session)
Significance levels are indicated by *p < 0.05, **p < 0.01, ***p < 0.001

Table R. Output summary for the self-reported stress

	Totally disagree (-3)	-2	-1	0	1	2	Totally agree (3)	Min	Median	Max
2700K	16	6	1	1	1	0	0	-3	-3	1
4000K	13	6	2	2	3	0	0	-3	-2.5	1
6500K	12	6	3	3	1	0	0	-3	-2	1
Total	*41*	*18*	*6*	*6*	*5*	*0*	*0*	*-3*	*-3*	*1*

Non-summary values represent the number of responses per category

Table S. Model summary for the self-reported stress

(Marginal model for correlated ordinal multinomial responses)

**Summary of residuals**					
Min.	1st quartile	Median	Mean	3rd quartile	Max.
-0.900	-0.168	-0.060	-0.001	-0.010	0.992

**Coefficients:**						
	Est/Beta	San.se	95% CI	San.z	p-value	Odds ratio
beta10	-1.996	1.199	-4.346 - 0.353	-1.665	0.096	ns
beta20	-0.493	1.064	-2.578 - 1.593	-0.463	0.643	ns
beta30	0.278	1.006	-1.693 - 2.249	0.276	0.782	ns
beta40	1.542	0.952	-0.324 - 3.409	1.620	0.105	ns
Pre-exposure	-0.729	0.144	-1.011 - -0.447	-5.060	<0.001***	0.480
CCT (4000K)	-0.612	0.525	-1.641 - 0.417	-1.166	0.244	ns
CCT (6500K)	-0.768	0.640	-2.023 - 0.487	-1.199	0.230	ns
Gender (female)	-0.423	0.559	-1.518 - 0.672	-0.757	0.449	ns
Age	0.034	0.018	-0.001 - 0.069	1.917	0.055	ns
Rain	-0.053	0.500	-1.032 - 0.926	-0.106	0.915	ns
Vision (indirect)	-0.811	0.510	-1.81 - 0.188	-1.591	0.112	ns
Street (Dorfstrasse Süd)	0.273	0.602	-0.907 - 1.452	0.453	0.651	ns
Street (Poststrasse)	0.415	0.780	-1.114 - 1.944	0.512	0.599	ns

Model equation: formula = Escape ~ LightColour + Gender + Age +Rain + Vision + Street , id = Session

Significance levels are indicated by *p < 0.05, **p < 0.01, ***p < 0.001, ns=non-significant

Table T. Output summary for the salivary cortisol

	Min	Median	Max	Mean	SD
**Pre-exposure**					
2700K	0.320	1.325	2.215	1.386	0.799
4000K	0.365	1.235	6.510	1.691	1.550
6500K	0.350	0.930	8.865	1.365	1.684
Total	*0.320*	*1.125*	*8.865*	*1.482*	*1.384*
**Post-exposure**					
2700K	0.410	1.195	2.720	1.267	0.586
4000K	0.395	1.075	3.725	1.321	0.809
6500K	0.275	0.795	3.725	1.048	1.049
Total	*0.275*	*1.01*	*5.59*	*1.214*	*0.830*

Table U. Model summary for the salivary cortisol
(linear mixed effect model).

Scaled residuals				
Min.	1st quartile	Median	3rd quartile	Max
-2.170	-0.715	-0.184	0.646	2.882

Fixed Effects					
	Est/Beta	SE	95% CI	t	p
Intercept	0.130	0.141	-0.15 - 0.41	0.922	0.360
Pre-exposure	0.570	0.023	0.525 - 0.616	24.822	<0.001***
CCT (4000K)	-0.109	0.075	-0.259 - 0.041	-1.448	0.152
CCT (6500K)	-0.182	0.076	-0.335 - -0.026	-2.388	0.020*
Gender (female)	0.094	0.064	-0.036 - 0.232	1.467	0.148
Age	0.005	0.002	0.001 - 0.009	2.555	0.013*
Rain	-0.133	0.070	-0.277 - 0.019	-1.895	0.080
Vision (indirect)	0.017	0.066	-0.114 - 0.147	0.257	0.798
Street (Dorfstrasse Süd)	0.165	0.080	-0.004 - 0.329	2.078	0.041*
Street (Poststrasse)	0.116	0.076	-0.043 - 0.271	1.524	0.132

Random Effects		
Groups	Variance	S.D.
Session (Intercept)	0.005	0.070
Residual	0.066	0.257

Model fit		
	Marginal	Conditional
R^2	0.897	0.904

Multiple comparisons: Tukey contrasts					
	Estimate	SE	95% CI	Z	p
4000K - 2700K	0.109	0.075	-0.285 - 0.067	1.448	0.296
6500K - 2700K	0.182	0.076	-0.36 - -0.003	2.388	0.051
6500K - 4000K	0.073	0.077	-0.253 - 0.107	0.953	0.341

P-values for fixed effects calculated using Satterthwaites approximations.
Model equation: Cortisol_T2 ~ Cortisol_T1 + LightColour + Gender + Age + Vision + Rain + Street + (1 | Session)

Significance levels are indicated by *p < 0.05, **p < 0.01, ***p < 0.001
